# Cost and clinical flow of point‐of‐care urine tenofovir testing for treatment monitoring among people living with HIV initiating ART in South Africa

**DOI:** 10.1002/jia2.70004

**Published:** 2025-07-14

**Authors:** Melody Wang, Pravikrishnen Moodley, Mlungisi Khanyile, Elliot Bulo, Makhosazane Zondi, Keshani Naidoo, Yukteshwar Sookrajh, Jienchi Dorward, Monica Gandhi, Nigel Garrett, Paul K. Drain, Monisha Sharma

**Affiliations:** ^1^ Department of Global Health University of Washington Seattle Washington USA; ^2^ National Health Laboratory Service (NHLS) Durban South Africa; ^3^ Centre for the AIDS Program of Research in South Africa (CAPRISA) University of KwaZulu‐Natal Durban South Africa; ^4^ eThekwini Municipality Health Unit eThekwini Municipality Durban South Africa; ^5^ Nuffield Department of Primary Care Health Sciences University of Oxford Oxford UK; ^6^ School of Medicine University of California San Francisco California USA; ^7^ Discipline of Public Health Medicine School of Nursing and Public Health University of KwaZulu‐Natal Durban South Africa; ^8^ Department of Medicine University of Washington Seattle Washington USA; ^9^ Department of Epidemiology University of Washington Seattle Washington USA

**Keywords:** antiretroviral therapy, clinic flow, cost analysis, drug adherence, point‐of‐care, South Africa, tenofovir

## Abstract

**Introduction:**

Point‐of‐care (POC) urine tenofovir (TFV) tests can provide timely information regarding antiretroviral therapy (ART) adherence to support management of HIV treatment in clinics. However, there are limited data on the costs and feasibility of integrating POC testing into HIV clinics in sub‐Saharan Africa. We characterized clinic flow and implementation costs of POC adherence testing for persons initiating ART in HIV care clinics in South Africa.

**Methods:**

We conducted a microcosting within a randomized controlled implementation trial of POC TFV test in government clinics in Durban, South Africa (STREAM HIV). Time‐and‐motion observation was conducted between 1st March and 31st December 2022, to assess staff and client time needed for POC TFV testing and counselling. We estimated both financial and economic costs for capital, clinic consumables and personnel using a provider (national government) perspective.

**Results:**

The estimated cost of POC TFV was USD $13 per client, assuming a clinic volume of 20 individuals initiating ART per month. The largest component costs of POC TFV testing were the test strip consumables, which accounted for 53% of the test cost. The median total time of a clinic visit with a POC TFV test, starting from client registration, was 49:19 (minutes: seconds) (IQR: 29:19–89:35). TFV testing took 9:22 (IQR: 7:35–14:11), taking up 19% of the total clinic visit time, including sample collection, sample loading, TFV test processing and counselling provision based on test results. Overall, 29% of the clinic visit time included direct clinical care and assessment with a provider, with clients spending a median 14:09 (IQR: 10:35–21:22) getting vitals checked, receiving adherence monitoring via POC TFV testing, and collecting their ART refill. Waiting in line for ART took most (48%) of the clinic visit time.

**Conclusions:**

POC TFV testing can be administered at reasonable costs, requires less than 10 minutes of healthcare provider time, and, therefore, may be feasible to implement in South African clinics. Findings can inform policy and budgetary planning for ART monitoring in South Africa and future cost‐effectiveness analyses of POC TFV testing.

**Clinical Trial Number:**

NCT04341779

## 1 INTRODUCTION

For the 28.7 million people living with HIV (PLHIV) accessing antiretroviral therapy (ART) globally in 2021, routine HIV viral load (VL) monitoring and sustained adherence to ART are both critical for viral suppression [1, 2]. Access to adherence testing and VL monitoring has been demonstrated to improve clinical outcomes for PLHIV, since identification of suboptimal ART adherence allows for timely clinical intervention, such as enhanced adherence counselling and medication changes [2, 3, 4, 5]. However, initiation and maintenance of lifelong ART in low‐ and middle‐income countries (LMICs) can be challenging for PLHIV. To assess adherence, providers mainly rely on subjective measures like self‐report, which can be unreliable and prone to recall error and bias [6, 7, 8]. Although objective assessment of ART adherence is possible through pharmacokinetic analysis of tenofovir (TFV) concentrations, broad adoption of pharmacologic measures is hindered by high cost, lengthy turnaround times and requirements for skilled personnel [9, 10]. Therefore, there is a demand for cost‐effective, time‐efficient and scalable approaches to assess drug adherence on ART [11, 12, 13, 14, 15].

A point‐of‐care (POC) test to assess TFV pill‐taking in urine has been developed and validated [16]. The test is a relatively inexpensive assay that has been developed to detect the presence of TFV with a short turnaround time in clinic‐based settings, which could complement more costly POC VL testing for monitoring [9, 17]. However, the costs of routinely administering these tests and the impact on clinic flow/provider time are uncertain [9, 17, 18, 19]. We sought to estimate the financial and economic costs of POC TFV testing for persons initiating ART in South Africa. To contextualize our findings to that of other POC tests, we updated previously estimated costs for POC VL testing from a pilot study conducted in 2017 in the same clinic [20]. Our research investigates the affordability of implementing POC adherence monitoring, and its sustainability within public healthcare clinics in South Africa.

## 2 METHODS

### 2.1 Study design

This study was embedded in the Simplifying Treatment & Monitoring for HIV (STREAM HIV) study (NCT04341779), a randomized controlled implementation trial designed to evaluate POC TFV and HIV VL testing in clinic settings in South Africa. Adult PLHIV initiating ART were randomized to receive either monthly POC TFV testing for 5 months and POC VL at 6 and 12 months or routine lab‐based monitoring as per the standard of HIV care in South Africa [18]. Demographic characteristics of the STREAM HIV study population are presented in Appendix S1. For the present study, we conducted a microcosting of urine‐based POC TFV testing from the provider perspective (Abbott Rapid Diagnostics Division, Orlando, Florida, USA). Activity‐based microcosting, staff interviews, and time and motion observations were conducted to estimate the costs of POC TFV testing. Interviews were conducted on‐site with providers, including clinicians, nurses and hospital administration, to understand workload and responsibilities. We estimated instrument costs assuming 5‐year lifespans with a 3% annual discount rate based on the WHO CHOICE guidelines and feedback from local clinic staff [21].

The STREAM HIV study builds upon the initial STREAM pilot study, in which our team estimated costs for implementing POC VL testing in 2017 [20, 22]. Thus, in addition to POC TFV microcosting, we also updated these POC VL testing costs to reflect 2022 costs using the gross domestic product price deflator and new prices for POC HIV VL test cartridges for the GeneXpert® platform (Cepheid, Sunnyvale, USA) [23, 24].

### 2.2 Study setting

Costing activities were conducted within two government clinics in KwaZulu‐Natal, South Africa. The urban Prince Cyril Zulu Communicable Disease Centre (CDC Clinic) government clinic is located adjacent to the CAPRISA eThekwini Clinical Research Site (ECRS), and provides primary care, sexual health, HIV and TB services. The CDC Clinic and ECRS are located near the transport hub for public commuters in central eThekwini (Durban), the largest city in the KwaZulu‐Natal province. The CDC Clinic initiates approximately 2000 PLHIV on ART annually. The rural Mafakathini Primary Healthcare Clinic is located in Vulindlela, adjacent to the CAPRISA Vulindlela Clinical Research Site (VCRS) and situated approximately 25 km from the provincial capital of KwaZulu‐Natal (Pietermaritzburg). Vulindlela is home to 400,000 residents, and the Mafakathini Clinic initiates approximately 180 PLHIV on ART annually.

### 2.3 Data sources and collection

We accessed budgets and expenditure records at health facilities. We used a more detailed ingredients approach to identify and value resources required for POC testing at the operational level, resulting in a comprehensive accounting of all inputs needed to deliver POC TFV testing.

Standardized cost menus were used to collect costs, including start‐up training, capital, clinic consumables and personnel time (Table 1). Staff salary and materials and instruments costs in 2022 US dollars were obtained from expense reports, clinic invoices, published government records, laboratory price lists, health facility staff and expert interviews, and health economics literature as needed. Notably, the POC TFV test strip is not currently available for commercial purchase, so only research use costs were calculated; the purchase price for routine use may vary when POC TFV testing becomes commercially available.

**Table 1:** Per‐test cost of point‐of‐care (POC) tenofovir (TFV) testing at varying clinic testing volumes in KwaZulu‐Natal, South Africa (2022 USD)

Cost category	Point‐of‐care costs by clinic testing volumes			
Tests conducted/month	10	20	50	100
Start‐up training	1.07	0.54	0.21	0.11
Capital costs	7.42	3.71	1.48	0.74
Clinic consumables	0.29	0.29	0.29	0.29
Test strip	6.86	6.86	6.86	6.86
Personnel time for testing and counselling	1.58	1.58	1.58	1.58
Total cost per test	17.22	12.97	10.43	9.58

*Note*: Total cost estimates assuming median personnel time for Professional Nurse cadre within a government clinic and 5% consumables wastage.

#### 2.3.1 Start‐up and capital costs

We estimated start‐up training costs from expense reports and interviews with trained staff; costs included salary for staff conducting and receiving the training and materials for the training workshop. Trainings were annualized using 5 years of useful life based on feedback from clinic staff and administration. We did not account for refresher trainings. Capital costs were obtained through expense reports and procurement plan contracts from the government CDC clinic.

#### 2.3.2 Material and consumables costs of POC VL and TFV testing

Materials were identified through direct observation and interviews with healthcare workers. Consumables for POC TFV testing included gloves, urine sample container, sticker labels and disposable pipette bulbs, and the unit research use cost of the POC TFV lateral flow test strip. We updated our prior microcosting of POC VL, adjusted for inflation and updated test cartridge costs [20]. See Appendix S2 for details on inflation adjustment.

#### 2.3.3 Time and Motion Study for POC TFV testing

Observed activities included: (1) clinical nurse time to collect urine; (2) processing and running POC tests; (3) post‐test adherence counselling based on results; and (4) completion of steps of the HIV care visit, including standard clinical assessment, addressing additional medical care queries and scheduling follow‐up visits. Professional Nurse health cadres, requiring a 4‐year degree and/or practical training programme, were observed. Lower‐level health cadres such as Enrolled Nurses, requiring a 2‐year diploma, and Enrolled Nurse Assistants, requiring 1 year of training, were not directly observed, but responsibilities and capacity for adherence testing were discussed during staff interviews.

To reduce sampling bias, participants were observed at different periods of the workday, days of the week and times of the year (March—May 2022 and November—December 2022). Data collectors observed a wide range of clients across clinical activities rather than observing fewer clients for their entire visit duration. Thus, if multiple clients were in the clinic at one time, data collectors would switch to and prioritize observing activities directly associated with the POC TFV testing flow over standard activities like patient registration. Clients with anomalous times for activities due to medical conditions needing urgent care were excluded from the analysis.

Staff time was enumerated by observing the total time needed for testing and removing non‐active waiting time during POC test processing as the staff completed routine clinic activities. Time needed for research activities, such as informed consent and reimbursements, were removed from time estimates to estimate intervention time if implemented within routine care in a government clinic. We estimated costs for active time staff for obtaining urine and blood samples, sample transport, test processing time, data record entry, test delivery to clients and clinical action if applicable.

### 2.4 Data analysis

Analyses followed the guidelines for costing trial interventions [25]. Cost categories included start‐up training, capital, clinic consumables and personnel salary required for POC TFV testing. Personnel costs were calculated by multiplying the staff salary per minute with the active time spent directly collecting the urine sample, running the test and counselling clients based on the test result (Appendix S3). Details on POC VL cost calculations are in Appendix S2.

To estimate total client volume, we referenced programmatic data from 59 clinics in eThekwini for our baseline assumption that a large clinic in South Africa would initiate 20 clients on ART per month [26]. We assumed the wastage of consumables for POC TFV testing to be 5% and used the median active personnel time needed to deliver the test in a clinic. We conducted sensitivity analyses varying ART clinic volume, cost of the POC TFV test strip, cadre of personnel conducting POC TFV testing and percent of wastage for clinic consumables.

### 2.5 Ethical approvals

Ethical approval was granted by the University of Washington Institutional Review Board (STUDY00007544) and the University of KwaZulu‐Natal Biomedical Research Ethics Committee (BREC/00000833/2019).

## 3 RESULTS

### 3.1 Costs per POC tests

We estimated that implementing POC TFV assays in public health clinics in South Africa cost USD $12.97 per test, assuming a clinic volume of 20 initiating ART clients per month (Table 1). Costs per client were substantially higher at lower testing volumes: $17.22 for clinics initiating 10 ART clients per month, versus $10.43 and $9.58 for clinics initiating 50 and 100 ART clients per month, respectively (Table 1).

Updated POC HIV VL costs were estimated at USD $29.74 per test, assuming a clinic volume of 20 initiating ART clients per month. Assuming a higher clinic volume of 50 initiating ART clients per month, POC HIV VL test costs decreased to $21.63 per test. The main cost driver for POC HIV VL testing was the $14.90 price of the consumable test cartridge, comprising 50% and 69% of the total cost, assuming a respective clinic volume of 20 and 50 ART initiations per month.

#### 3.1.1 Sensitivity analyses

**Figure 1:**
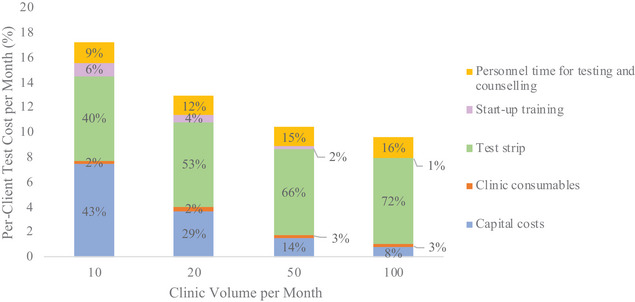
Percentage of total point‐of‐care (POC) tenofovir (TFV) costs per category, varying monthly clinic testing volumes for antiretroviral therapy (ART) initiations (2022 USD).

**Figure 2:**
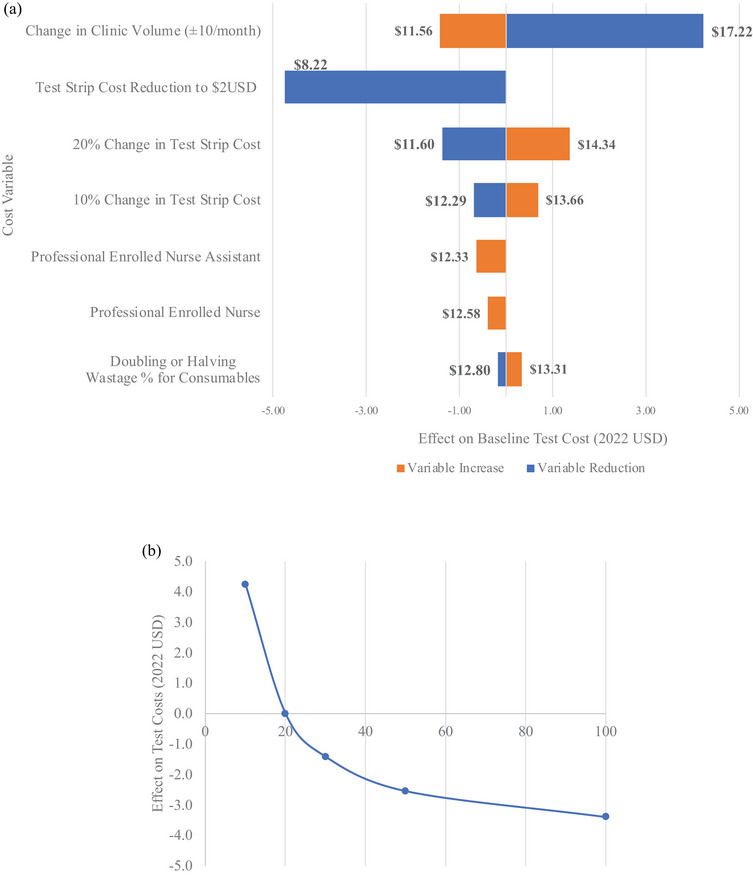
(A) Sensitivity analysis assessing potential impacts of varying cost assumptions with bars displaying changes in US dollars (USD) from the total baseline point‐of‐care (POC) tenofovir (TFV) cost. Variable total costs were compared against a baseline per‐client cost of POC TFV test that assumed 20 antiretroviral therapy (ART) clients initiated per month, median personnel time, professional nurse cadre conducting the test, 5% wastage of clinic consumables and cost of test strip procured for the intervention. (B) Effect of monthly antiretroviral therapy (ART) client volume changes on total point‐of‐care (POC) tenofovir (TFV) test costs. Per‐client cost of POC TFV test using baseline assumptions of median personnel time, professional nurse cadre conducting the test, 5% wastage of clinic consumables and cost of test strip procured for the intervention were calculated at varying monthly clinic volumes of 10, 20, 30, 50 and 100 clients initiated on ART.

The largest contributor to the cost per test was the POC TFV test strip (estimated at $6.86), which was 53% of the total cost for urine TFV testing and counselling (Figure 1). Sensitivity analyses showed that changes in monthly ART clinic volumes and POC TFV test strip price had a substantial impact on the total cost (Figure 2A). For example, a 20% and 10% reduction in the current POC TFV test strip cost resulted in per‐client test cost of $11.60 and $12.29, respectively. As commercial assays become routinely available, test strip prices may decrease to $2, which would result in a 37% reduction in per‐client test costs to $8.22 (Figure 2A). Conversely, a 20% and 10% increase in the test strip cost would increase per‐client test costs to $14.34 and $13.66. Reducing personnel costs by task shifting to Enrolled Nurses and Enrolled Nursing Assistants decreased the POC TFV cost per‐test by just 3% and 5%, respectively. Varying wastage costs also had a minimum impact on results (Figure 2A), as did median personnel time needed for testing and using a 10‐year time horizon (Appendix S4). Varying assumptions of monthly ART clinic volumes were modelled in Figure 2B and showed diminishing returns in cost reductions for total POC TFV testing after ART clinic volume increased by more than 50 clients/month.

### 3.2 Clinic flow for POC adherence monitoring

**Figure 3:**
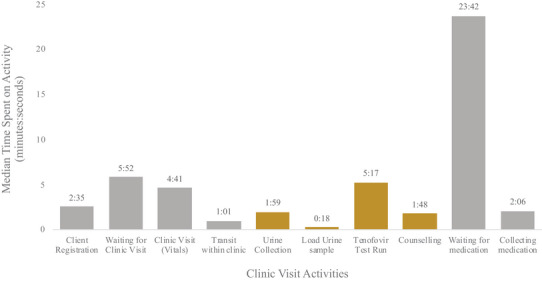
Clinical flow showing median time (minutes:seconds) (y‐axis) participant spent during each activity type (x‐axis) during a complete clinic visit including objective adherence monitoring. Activities specific for the point‐of‐care (POC) urine‐based tenofovir (TFV) test process are shown in yellow.

Clinical flow and times for visits, including POC TFV testing, are presented in Figure 3 and Figure S1. Clinic staff arrive between 6:30 AM to 7:00 AM and stay until 4:00 PM. Clinics open at 8 AM, with most clients arriving earlier to queue and most clients are seen in the mornings until lunchtime (1:00 PM). Following collection of their chart, the median time clients spent in the clinic for a visit with a POC TFV test was 49:19 (minutes: seconds) (interquartile range [IQR]: 29:19–89:35). The POC TFV testing took a median time of 9:22 (IQR: 7:35–14:11) (19% of total clinic visit) including sample collection, sample loading, TFV test processing and counselling provision based on test results (Figure 3). Urine collection was observed among 38 clients, with the median time of sample collection being 1:59 (IQR: 1.28–2.30). Among 15 samples of urine, the median time to load a plastic pipette and deposit three drops onto the POC TFV test strip was 0:18 (IQR: 0.14–0.46). The median time for running a POC TFV test was 5:17 (IQR: 5:00–5:36) among 32 observations. All test results observed were positive for TFV, so counselling was only observed for adherent POC TFV test results among 28 clients, with a median active time of 1:48 (IQR: 0:54–5:19). Sensitivity analyses did not find significant differences in POC TFV testing times by sex.

### 3.3 Client experience and direct interaction with providers

Overall, only 29% of the clinic visit time included direct clinical care and assessment with a provider; clients spent a median time of 14:09 (IQR: 10:35–21:22) getting vitals checked, adherence monitored via POC TFV testing and collecting ART medication. Clients spent 11% (5:35 [IQR: 4:00–7:43]) of their clinic visit registering at the front desk, walking within the clinic and providing urine samples, and 60% (29:35 [IQR: 14:44–60:30]) of their clinic visit waiting for clinical attention, either for measuring vitals or collecting ART medication refills.

Waiting in line for ART took most (48%) of the clinic visit time (median time of 23:42, IQR: 12:03–38:13, *n* = 32). Waiting for clinical vital measurement took a shorter median time of 5:52 (IQR: 2:41–22:17, *n* = 16). Median clinical appointment duration with the ART nurse was only 2:06 (IQR: 1:38–3:21, *n* = 25) after the wait. Checking for clinic vitals, including measuring client blood pressure, body weight and waist circumference, took a median time of 4:41 (IQR: 2:50–6:20, *n* = 40). Personnel shortages and inefficient clinical flow between the POC TFV testing and the ART queue were noted by staff as potential areas for improvement for the future.

## 4 DISCUSSION

We estimated the costs of implementing POC TFV testing for drug adherence monitoring in South Africa. The per‐client test costs for POC TFV were less than half of POC HIV VL testing. The primary cost drivers for both POC tests were clinic consumables, specifically the test strip for TFV and the test cartridge for VL. POC TFV testing costs were sensitive to ART client volumes in the clinic, which is in line with prior POC HIV VL costing analyses, as well as the TFV test strip price [20, 27]. POC TFV testing flow required less than 10 minutes, and was much faster than the queues experienced by clients collecting standard ART refills.

A 2019 systematic review of costs of HIV services in South Africa found that more cost analyses of non‐ART interventions were needed, particularly for adherence monitoring [28]. The POC TFV testing costs and clinical flow presented in this study are the first reported in a routine implementation setting, so there is a limited literature within which to contextualize our findings. POC VL test costs are consistent with other studies, with the estimated per‐test cost of POC HIV VL testing ranging from USD $24.25 per test up to $33.71 when using the Cepheid GeneXpert four‐module testing platform [20, 27, 29, 30]. Costing studies of POC CD4, creatinine and urine lipoarabinomannan testing in South Africa have estimated per‐client costs to be USD $11, $9 and $12, respectively [20, 31]. Compared to these diagnostics, the estimated cost of POC TFV testing in our study (∼$13 USD per test) is within a similar range to existing POC technologies. Particularly in resource‐limited settings, variable assumptions on clinic infrastructure may also contribute to different cost estimations [13, 14, 27].

Though there are limited costing analyses for POC TFV testing, qualitative studies exploring perspectives on the POC urine TFV test among clients and providers in the United States reported favourable acceptability among providers when used in tandem with VL testing [10]. Qualitative results concluded that POC TFV tests should be low‐cost, akin to the price of a urine‐based pregnancy test, in order to support widespread use [10]. Evaluation of urine‐based POC TFV assay implementation in South Africa reported positive perceptions from PLHIV regarding the ability to have constructive real‐time discussions with providers, while providers noted that POC TFV testing could help identify clients at risk of treatment failure [32, 33]. Healthcare workers also noted the importance of additional support staff for successful implementation in high‐volume health facilities [10, 32]. Given the minimal cost difference found between staff levels, task‐shifting may not yield substantial financial benefits; however, it may still be helpful if there are higher‐skilled personnel shortages. POC technologies can accelerate adherence support and VL monitoring to create new integrated models of person‐centred care in LMICs. However, incorporating them without sufficient evidence regarding impact on clinics may overwhelm existing clinical flows and exacerbate waiting times in resource‐limited healthcare facilities.

Our results are consistent with the literature showing that consumables make up a significant component of test costs [20, 27, 34]. Addressing high POC testing consumable costs may contribute to impactful reductions in total POC testing costs for national programmes. This can include negotiations with manufacturers to lower pricing for test cartridges, as well as greater integration on multi‐disease testing platforms to enable cost‐sharing between disease programmes [27, 35]. Using research use costs for the POC TFV assay, we found that total test costs were sensitive to fluctuations in test strip consumable prices. Routine use costs for the POC TFV assay are currently estimated to be less than one‐third of the research use cost, at USD $2 per test [36]. This could significantly lower total POC TFV testing costs and improve access to adherence monitoring. Continued global engagement with manufacturers is needed to ensure sustainable prices for this technology if resource‐limited countries are to consider the implementation of POC TFV testing.

The study has several limitations. First, our analysis was conducted within a randomized control trial, which may not reflect routine implementation [37]. However, to maximize external validity, the STREAM HIV trial was designed so that the study population was representative of the population and clinical settings in which POC adherence testing will be implemented [18, 38]. Following best practices, we removed research‐related costs and only accounted for active personnel time delivering POC TFV testing services, reflecting real‐world implementation in a government programme [25, 37, 39]. Second, the collection of time and motion data may be influenced by the Hawthorne effect, in which participants’ behaviours change due to observation [40]. To address this, we informed participants that observation was not for worker performance evaluation and observations were conducted at varying times throughout the day, week and across multiple seasons to improve accuracy and allow for providers to settle into normal work patterns. Additionally, since the vast majority (99%) of clients were adherent to TFV in the parent study, we did not observe any negative test results and, therefore, cannot assess their impact on provider time.

Lastly, implementation costs and activity time could vary by location. We aimed to collect data across multiple staff members and from both urban and rural clinic locations in KwaZulu‐Natal to account for variations. However, due to the limited number of clients available to observe at the rural clinic, a comparative analysis between the urban and rural clinics was not possible. Staff interviews were conducted at both urban and rural clinics to increase the understanding of site‐level variation for POC TFV sensitivity analyses parameters and interpretation of results. Further qualitative and costing studies should be conducted to determine the feasibility and acceptability of implementing the POC TFV adherence test in different contexts. Additionally, our cost estimates can be used in future cost‐effectiveness analyses of POC TFV testing for adherence monitoring.

In addition to its use for adherence monitoring of ART clients, POC TFV testing also has potential utility for encouraging adherence, particularly among young women, to pre‐exposure prophylaxis (PrEP) and as a predictive tool for detecting HIV drug resistance (HIVDR) to TFV‐containing ART regimens [5, 41, 42, 43]. Consistent use of PrEP is vital for key populations with PrEP indication in order to achieve “prevention‐effective” adherence, which has been shown to be modest and declining over time [41]. Studies in Kenya have shown that real‐time measures of PrEP adherence using a POC urine test, as opposed to more invasive and costly objective measures, were feasible in the context of routine care, acceptable to clients and providers, and could improve adherence [42]. Genotypic DR testing for HIV is not commonly available in LMICs; thus, treatment decisions after repeated elevated VL results often follow standardized national clinical guidelines without any prior DR testing [43, 44]. Among people with viraemia, a positive urine TFV test may be associated with the presence of HIVDR [5, 45, 46], but findings have been mixed [47]. Targeted POC TFV reflex testing following elevated VL results could also highlight the need for intensive adherence counselling. Thus, our study findings contribute practical evidence on the use of a low‐cost, urine‐based diagnostic tool that has the potential to not only support ART adherence among PLHIV, but also key populations receiving PrEP to prevent HIV acquisition and people with potential DR [5, 43]. Widespread availability of objective adherence monitoring tools with lower time and cost barriers, such as POC TFV testing, has the potential to streamline adherence support, complement existing treatment monitoring strategies and expand HIV service delivery beyond clinical settings to support HIV differentiated care models.

## 5 CONCLUSIONS

In summary, our microcosting and Time and Motion Study analyses demonstrate that POC TFV testing could be feasibly implemented into routine care at a reasonable cost, particularly within clinics with higher ART client volumes and if the test is available commercially at a price similar to or lower than the research cost. POC TFV testing is straightforward, has minimal infrastructure requirements and is not a substantial time burden to incorporate into the overall clinical workflow. In addition to aiding drug adherence among PLHIV, our results may also encourage the use of POC TFV testing as inexpensive complementary diagnostic tools to support PrEP adherence and HIVDR testing. Our findings can inform the policy decisions and budgetary planning for ART monitoring in South Africa and other LMICs, considering the implementation of POC TFV and VL testing as alternatives to traditional laboratory‐based testing. Results can be used in economic evaluations to assess the cost‐effectiveness of POC TFV testing for adherence monitoring to complement other diagnostic technologies.

## COMPETING INTERESTS

The authors declare that they have no competing interests.

## AUTHORS’ CONTRIBUTIONS

All authors contributed to the study and met the criteria for authorship. PKD, NG, MG and MS conceived the study. Methodology was determined by MS and MW. Data collected thanks to MW, MZ, PM, MK, EB, KN and YS. Data analysis and interpretation conducted by MW and MS, then reviewed by PKD, JD, NG and MG. MW wrote the original draft of the manuscript, and all authors reviewed, edited and approved the final manuscript.

## FUNDING

This project was funded by the National Institute of Allergy and Infectious Diseases (R01AI147752) and the University of Washington.

## Supporting information




**Table S1**: Per client point‐of‐care (POC) tenofovir (TFV) cost component quantities and prices.
**Figure S1**: Clinic flow diagram for healthcare clients during a clinic visit, including point‐of‐care (POC) tenofovir (TFV) testing and counselling. Following client registration (Step 1) in the clinical records room, the client moved to the waiting area for the clinical room (Step 2). Following the clinic visit checking client vitals (Step 3), the client would transit within the clinic (Step 4) to collect their urine sample (Step 5). Note that transit times between registration and clinic spaces, to and from an on‐site washroom (WC), and to collect medication were all combined for the analysis. Following urine collection, the client would move to a separate private area in the back of the clinical space for staff to load the urine sample (Step 6), run the POC TFV test (Step 7) and provide counselling (Step 8). Clients would then head to the designated area to wait for antiretroviral therapy (ART) medication collection (Step 9). Following the collection of medication (Step 10), the client would then conclude their clinic visit and exit the facility.

**Appendix S1**: Socio‐demographic characteristics of the parent study (STREAM HIV) cohort at baseline (*N* = 539).
**Appendix S2**: Gross domestic product (GDP) price deflator methods for updating point‐of‐care (POC) viral load (VL) testing cost estimates.
**Appendix S3**: Personnel time and costs.
**Appendix S4**:: Additional sensitivity analyses.

## Data Availability

Data collected and analysed to support the findings of this study are available on request from the corresponding author. The data are not publicly available due to privacy restrictions.

## References

[1] UNAIDS . UNAIDS Global HIV Statistics Fact Sheet. 2022.

[2] WHO . The role of HIV viral suppression in improving individual health and reducing transmission. 2023.

[3] Myer L , Essajee S , Broyles LN , Watts DH , Lesosky M , El‐Sadr WM , et al. Pregnant and breastfeeding women: a priority population for HIV viral load monitoring. PLoS Med. 2017;14(8):e1002375. 28809929 10.1371/journal.pmed.1002375 PMC5557351

[4] Moosa A , Gengiah TN , Lewis L , Naidoo K . Long‐term adherence to antiretroviral therapy in a South African adult patient cohort: a retrospective study. BMC Infect Dis. 2019;19(1):775. 31488063 10.1186/s12879-019-4410-8 PMC6727323

[5] McCluskey SM , Govender K , Adamson J , Gandhi M , Spinelli MA , Moosa M‐Y , et al. Point‐of‐care urine tenofovir testing to predict HIV drug resistance among individuals with virologic failure. AIDS. 2023;37(7):1109–1113. 36928169 10.1097/QAD.0000000000003520 PMC10164085

[6] Orrell C , Cohen K , Leisegang R , Bangsberg DR , Wood R , Maartens G . Comparison of six methods to estimate adherence in an ART‐naive cohort in a resource‐poor setting: which best predicts virological and resistance outcomes? AIDS Res Ther. 2017;14(1):20. 28376815 10.1186/s12981-017-0138-y PMC5379739

[7] Simoni JM , Kurth AE , Pearson CR , Pantalone DW , Merrill JO , Frick PA . Self‐report measures of antiretroviral therapy adherence: a review with recommendations for HIV research and clinical management. AIDS Behav. 2006;10(3):227–245. 16783535 10.1007/s10461-006-9078-6 PMC4083461

[8] Stirratt MJ , Dunbar‐Jacob J , Crane HM , Simoni JM , Czajkowski S , Hilliard ME , et al. Self‐report measures of medication adherence behavior: recommendations on optimal use. Transl Behav Med. 2015;5(4):470–482. 26622919 10.1007/s13142-015-0315-2 PMC4656225

[9] Gandhi M , Bacchetti P , Rodrigues WC , Spinelli M , Koss CA , Drain PK , et al. Development and validation of an immunoassay for tenofovir in urine as a real‐time metric of antiretroviral adherence. EClinicalMedicine. 2018;2–3:22–28. 10.1016/j.eclinm.2018.08.004 PMC6428441 30906930

[10] Bardon AR , Simoni JM , Layman LM , Stekler JD , Drain PK . Perspectives on the utility and interest in a point‐of‐care urine tenofovir test for adherence to HIV pre‐exposure prophylaxis and antiretroviral therapy: an exploratory qualitative assessment among U.S. clients and providers. AIDS Res Ther. 2020;17(1):50. 32762713 10.1186/s12981-020-00308-w PMC7412814

[11] Lecher S , Ellenberger D , Kim AA , Fonjungo PN , Agolory S , Borget MY , et al. Scale‐up of HIV viral load monitoring — seven sub‐Saharan African countries. MMWR Morb Mortal Wkly Rep. 2015;64:1287–1290. 26605986 10.15585/mmwr.mm6446a3

[12] Ndlovu Z , Fajardo E , Mbofana E , Maparo T , Garone D , Metcalf C , et al. Multidisease testing for HIV and TB using the GeneXpert platform: a feasibility study in rural Zimbabwe. PLoS One. 2018;13(3):e0193577. 29499042 10.1371/journal.pone.0193577 PMC5834185

[13] Elsbernd K , Emmert‐Fees KMF , Erbe A , Ottobrino V , Kroidl A , Bärnighausen T , et al. Costs and cost‐effectiveness of HIV early infant diagnosis in low‐ and middle‐income countries: a scoping review. Infect Dis Poverty. 2022;11(1):82. 35841117 10.1186/s40249-022-01006-7 PMC9284833

[14] WHO . Systematic review of HIV testing costs in high and low income settings. 2015.

[15] Wu G , Zaman MH . Low‐cost tools for diagnosing and monitoring HIV infection in low‐resource settings. Bull World Health Organ. 2012;90(12):914–920. 23284197 10.2471/BLT.12.102780 PMC3524957

[16] Gandhi M , Bacchetti P , Spinelli MA , Okochi H , Baeten JM , Siriprakaisil O , et al. Brief report: validation of a urine tenofovir immunoassay for adherence monitoring to PrEP and ART and establishing the cutoff for a point‐of‐care test. J Acquir Immune Defic Syndr. 2019;81(1):72–77. 30664078 10.1097/QAI.0000000000001971 PMC6456396

[17] Gandhi M , Wang G , King R , Rodrigues WC , Vincent M , Glidden DV , et al. Development and validation of the first point‐of‐care assay to objectively monitor adherence to HIV treatment and prevention in real‐time in routine settings. AIDS. 2020;34(2):255–260. 31634188 10.1097/QAD.0000000000002395 PMC7021226

[18] Bardon AR , Dorward J , Sookrajh Y , Sayed F , Quame‐Amaglo J , Pillay C , et al. Simplifying TREAtment and Monitoring for HIV (STREAM HIV): protocol for a randomised controlled trial of point‐of‐care urine tenofovir and viral load testing to improve HIV outcomes. BMJ Open. 2021;11(10):e050116. 10.1136/bmjopen-2021-050116 PMC8493905 34610939

[19] Drain PK , Dorward J , Violette LR , Quame‐Amaglo J , Thomas KK , Samsunder N , et al. Point‐of‐care HIV viral load testing combined with task shifting to improve treatment outcomes (STREAM): findings from an open‐label, non‐inferiority, randomised controlled trial. Lancet HIV. 2020;7(4):e229–e237. 32105625 10.1016/S2352-3018(19)30402-3 PMC7183312

[20] Simeon K , Sharma M , Dorward J , Naidoo J , Dlamini N , Moodley P , et al. Comparative cost analysis of point‐of‐care versus laboratory‐based testing to initiate and monitor HIV treatment in South Africa. PLoS One. 2019;14(10):e0223669. 31618220 10.1371/journal.pone.0223669 PMC6795460

[21] Bertram MY , Edejer TTT . Introduction to the Special Issue on “The World Health Organization Choosing Interventions That Are Cost‐Effective (WHO‐CHOICE) Update”. Int J Health Policy Manag. 2021;10(11):670–672. 34634892 10.34172/ijhpm.2021.105 PMC9278374

[22] Dorward J , Garrett N , Quame‐Amaglo J , Samsunder N , Ngobese H , Ngomane N , et al. Protocol for a randomised controlled implementation trial of point‐of‐care viral load testing and task shifting: the Simplifying HIV TREAtment and Monitoring (STREAM) study. BMJ Open. 2017;7(9):e017507. 10.1136/bmjopen-2017-017507 PMC5623564 28963304

[23] Chowdhury A . Methods explained: the GDP implied deflator. Econ Labour Market Rev. 2008;2:53–56.

[24] Church JD . Comparing the consumer price index with the gross domestic product price index and gross domestic product implicit price deflator. Month Labor Rev. 2016;139:1.

[25] Vassall A , Sweeney S , Kahn J , Gomez G , Bollinger L , Marseille E , et al. Reference case for estimating the costs of global health services and intervention. 2017.

[26] Dorward J , Sookrajh Y , Khubone T , van der Molen J , Govender R , Phakathi S , et al. Implementation and outcomes of dolutegravir‐based first‐line antiretroviral therapy for people with HIV in South Africa: a retrospective cohort study. Lancet HIV. 2023;10(5):e284–e294. 37001536 10.1016/S2352-3018(23)00047-4 PMC10288006

[27] Bulterys MA , Oyaro P , Brown E , Yongo N , Karauki E , Wagude J , et al. Costs of point‐of‐care viral load testing for adults and children living with HIV in Kenya. Diagnostics (Basel). 2021;11(1):140. 33477850 10.3390/diagnostics11010140 PMC7832863

[28] Meyer‐Rath G , van Rensburg C , Chiu C , Leuner R , Jamieson L , Cohen S . The per‐patient costs of HIV services in South Africa: systematic review and application in the South African HIV Investment Case. PLoS One. 2019;14(2):e0210497. 30807573 10.1371/journal.pone.0210497 PMC6391029

[29] Girdwood SJ , Crompton T , Sharma M , Dorward J , Garrett N , Drain PK , et al. Cost‐effectiveness of adoption strategies for point of care HIV viral load monitoring in South Africa. EClinicalMedicine. 2020;28:100607. 33294817 10.1016/j.eclinm.2020.100607 PMC7700965

[30] Ganesh P , Heller T , Chione B , Gumulira J , Gugsa S , Khan S , et al. Near point‐of‐care HIV viral load: targeted testing at large facilities. J Acquir Immune Defic Syndr. 2021;86(2):258–263. 33136821 10.1097/QAI.0000000000002555 PMC7803448

[31] Mukora R , Tlali M , Monkwe S , Charalambous S , Karat AS , Fielding KL , et al. Cost of point‐of‐care lateral flow urine lipoarabinomannan antigen testing in HIV‐positive adults in South Africa. Int J Tuberc Lung Dis. 2018;22(9):1082–1087. 30092876 10.5588/ijtld.18.0046 PMC6086286

[32] McInziba A , Wademan D , Viljoen L , Myburgh H , Jennings L , Decloedt E , et al. Perspectives of people living with HIV and health workers about a point‐of‐care adherence assay: a qualitative study on acceptability. AIDS Care. 2023;35(10):1628–1634. 36781407 10.1080/09540121.2023.2174928 PMC10423296

[33] Proctor E , Silmere H , Raghavan R , Hovmand P , Aarons G , Bunger A , et al. Outcomes for implementation research: conceptual distinctions, measurement challenges, and research agenda. Adm Policy Ment Health. 2011;38(2):65–76. 20957426 10.1007/s10488-010-0319-7 PMC3068522

[34] Larson B , Schnippel K , Ndibongo B , Long L , Fox MP , Rosen S . How to estimate the cost of point‐of‐care CD4 testing in program settings: an example using the Alere Pima Analyzer in South Africa. PLoS One. 2012;7(4):e35444. 22532854 10.1371/journal.pone.0035444 PMC3331987

[35] Wang M , Boeke CE , Rioja MR , Maparo T , Banda C , Chavula C , et al. Feasibility and impact of near‐point‐of‐care integrated tuberculosis/HIV testing in Malawi and Zimbabwe. AIDS. 2021;35(15):2531–2537. 34310372 10.1097/QAD.0000000000003031

[36] Gandhi M . Personal Communication. 2024.

[37] Petrou S , Gray A . Economic evaluation alongside randomised controlled trials: design, conduct, analysis, and reporting. BMJ. 2011;342:d1548. 21474510 10.1136/bmj.d1548 PMC3230107

[38] Ramsey SD , Willke RJ , Glick H , Reed SD , Augustovski F , Jonsson B , et al. Cost‐effectiveness analysis alongside clinical trials II‐an ISPOR Good Research Practices Task Force report. Value Health. 2015;18(2):161–172. 25773551 10.1016/j.jval.2015.02.001

[39] Curran GM , Landes SJ , McBain SA , Pyne JM , Smith JD , Fernandez ME , et al. Reflections on 10 years of effectiveness‐implementation hybrid studies. Front Health Serv. 2022;2:1053496. 36925811 10.3389/frhs.2022.1053496 PMC10012680

[40] McCambridge J , Witton J , Elbourne DR . Systematic review of the Hawthorne effect: new concepts are needed to study research participation effects. J Clin Epidemiol. 2014 Mar 1;67(3):267–277. 24275499 10.1016/j.jclinepi.2013.08.015 PMC3969247

[41] Haberer JE , Mugo N , Bukusi EA , Ngure K , Kiptinness C , Oware K , et al. Understanding pre‐exposure prophylaxis adherence in young women in Kenya. J Acquir Immune Defic Syndr. 2022;1999(89):251. 10.1097/QAI.0000000000002876 PMC8826617 35147580

[42] Thuo N , Polay M , Leddy AM , Ngure K , Chatterhee P , Gandhi M , et al. Point‐of‐care test for assessing tenofovir adherence: feasibility and recommendations from women in an oral PrEP program in Kenya and their healthcare providers. AIDS Behav. 2021;25(11):3617–3629. 33893877 10.1007/s10461-021-03255-3 PMC9271229

[43] Zaccarelli M , Niyongabo B , Conway B . Point‐of‐care urine tenofovir testing: making a good thing better. AIDS. 2023;37(7):1159–1160. 37139650 10.1097/QAD.0000000000003539

[44] Health RoSANDo . ART clinical guidelines for the management of HIV in adults, pregnancy, adolescents, children, infants, and neonates. National Department of Health; 2020.

[45] Hermans LE , Umunnakwe CN , Lalla‐Edward ST , Hebel SK , Tempelman HA , Nijhuis M , et al. Point‐of‐care tenofovir urine testing for the prediction of treatment failure and drug resistance during initial treatment for human immunodeficiency virus type 1 (HIV‐1) infection. Clin Infect Dis. 2023;76(3):e553–e560. 36136811 10.1093/cid/ciac755 PMC9907515

[46] Martinson T , Nwogu‐Attah J , Spinelli M , Gandhi M . Low‐cost urine tenofovir assay to triage dolutegravir resistance testing. Lancet HIV. 2024;11(5):e282–e283. 38461845 10.1016/S2352-3018(24)00060-2 PMC11663141

[47] Dorward J , Lessells R , Govender K , Moodley P , Samsunder N , Sookrajh Y , et al. Diagnostic accuracy of a point‐of‐care urine tenofovir assay, and associations with HIV viraemia and drug resistance among people receiving dolutegravir and efavirenz‐based antiretroviral therapy. medRxiv. 2023:2023–2004. 10.1002/jia2.26172 PMC10514373 37735860

[48] World Bank . World Development Indicators: Exchange rates and prices [Internet]. 2023 [cited 2024 Jul 19]. Available from: https://wdi.worldbank.org/table/4.16

