# Supplementary material for: Cost and clinical flow of point‐of‐care urine tenofovir testing for treatment monitoring among people living with HIV initiating ART in South Africa
Source: J Int AIDS Soc. 2025 Jul 14;28(7):e70004. doi: 10.1002/jia2.70004 (PMC12260116; doi:10.1002/jia2.70004)
Supplement: Supplementary file 1 — Table S1: Per client point‐of‐care (POC) tenofovir (TFV) cost component quantities and prices. Figure S1: Clinic flow diagram for healthcare clients during a clinic visit, including point‐of‐care (POC) tenofovir (TFV) testing and counselling. Following client registration (Step 1) in the clinical records room, the client moved to the waiting area for the clinical room (Step 2). Following the clinic visit checking client vitals (Step 3), the client would transit within the clinic (Step 4) to collect their urine sample (Step 5). Note that transit times between registration and clinic spaces, to and from an on‐site washroom (WC), and to collect medication were all combined for the analysis. Following urine collection, the client would move to a separate private area in the back of the clinical space for staff to load the urine sample (Step 6), run the POC TFV test (Step 7) and provide counselling (Step 8). Clients would then head to the designated area to wait for antiretroviral therapy (ART) medication collection (Step 9). Following the collection of medication (Step 10), the client would then conclude their clinic visit and exit the facility. [file JIA2-28-e70004-s001.docx]

**SUPPLEMENTARY MATERIALS**

Table S1. Per client point-of-care (POC) tenofovir (TFV) cost component quantities and prices.

|  | Quantity | Unit Cost (USD) | Total Cost for Component (USD) |
| --- | --- | --- | --- |
| Start-up Training^1^ | 1 | 10.71 | 0.54 |
| Capital Costs^2^ | | | 3.71 |
| Table to process testing and sit with clients | 0.1 | 0.13 |  |
| Plastic chair for patient | 1 | 0.07 |  |
| Generators | 0.015 | 2.88 |  |
| Testing Space (square meters)^3^ | 3 | 18.98 |  |
| Water and Electricity Bill | 0.015 | 24.60 |  |
| Pen (Black ballpoint pen) | 12 | 0.28 |  |
| Toilet installation and maintenance, hand paper towel and soap dispensers for all consulting rooms | 0.015 | 5.27 |  |
| Healthcare risk waste storage | 0.015 | 21.97 |  |
| Clinic Consumables^4^ | | | 0.29 |
| Sticker label for urine sample | 1 | 0.02 |  |
| Gloves | 2 | 0.18 |  |
| Urine sample container | 1 | 0.09 |  |
| Pipette bulb (included in the POC TFV test kit) | 1 | N/A |  |
| POC TFV Test Strip^4^ | 1 | 6.53 | 6.86 |
| Personnel to provide POC TFV testing (minutes:seconds)^5^ | 7.37 | 0.21 | 1.58 |
| Total Test Cost | | | 12.97 |

^1^ Start-up training costs included practice test strips and one full-day (8 hours) salary to compensate both the trainer and healthcare workers for their time. Start-up training costs were then annualized and presented as cost per month.

^2^ Capital costs were annualized assuming a 5-year lifespan at 3% annual discount rate, as well as a clinic volume of 20 antiretroviral therapy (ART) clients per month. Quantity of capital costs were estimated using the 2022-2023 procurement plan and budget obtained from local government hospital administration.

^3^ Testing space needs based surface area needed for standard table, chair, and personnel. Unit costs estimated by pro-rating the average rental cost per square meter in the region.

^4^ Quantity of clinic consumables was based on observation of standard POC TFV test process. Wastage of 5% of consumables was incorporated into to the final cost estimate. Quality control strips for POC TFV testing were also incorporated into clinic consumable costs.

^5^ Personnel time was estimated using time and motion analysis, and median time was determined to be 7:37 (minutes:seconds) (interquartile range [IQR]: 6:07 – 11:41). Official government clinic salary for a Professional Nurse healthcare worker cadre is 12.86 USD per hour, which translates to 21 cents per minute.

Figure S1. Clinic flow diagram for healthcare clients during a clinic visit including point-of-care (POC) tenofovir (TFV) testing and counselling. Following client registration (Step 1) in the clinical records room, the client moved to the waiting area for the clinical room (Step 2). Following the clinic visit checking client vitals (Step 3), the client would transit within the clinic (Step 4) to collect their urine sample (Step 5). Note that transit times between registration and clinic spaces, to and from an on-site washroom (WC), and to collect medication were all combined for the analysis. Following urine collection, the client would move to a separate private area in the back of the clinical space for staff to load the urine sample (Step 6), run the POC TFV test (Step 7) and provide counselling (Step 8). Clients would then head to the designated area to wait for antiretroviral therapy (ART) medication collection (Step 9). Following collection of medication (Step 10), the client would then conclude their clinic visit and exit the facility.
