# Supplementary material for: Cost and clinical flow of point‐of‐care urine tenofovir testing for treatment monitoring among people living with HIV initiating ART in South Africa
Source: J Int AIDS Soc. 2025 Jul 14;28(7):e70004. doi: 10.1002/jia2.70004 (PMC12260116; doi:10.1002/jia2.70004)
Supplement: Supplementary file 2 — Appendix S1: Socio‐demographic characteristics of the parent study (STREAM HIV) cohort at baseline (N = 539). Appendix S2: Gross domestic product (GDP) price deflator methods for updating point‐of‐care (POC) viral load (VL) testing cost estimates. Appendix S3: Personnel time and costs. Appendix S4:: Additional sensitivity analyses. [file JIA2-28-e70004-s002.docx]

**APPENDICES**

**Appendix 1.** Sociodemographic characteristics of the parent study (STREAM HIV) cohort at baseline (N=539).

| **Sociodemographic Characteristics** | **n (% Total)** |
| --- | --- |
| Age (years) (Mean, SD^1^) | 33.4 ± 8.5 |
| Sex |  |
| Female | 317 (58.8%) |
| Male | 222 (41.2%) |
| Ethnicity |  |
| Black | 521 (96.7%) |
| Other | 18 (3.3%) |
| Highest level of education |  |
| None | 1 (0.2%) |
| Primary school | 23 (4.3%) |
| Passed matric | 187 (34.7%) |
| Did not pass matric | 237 (44%) |
| Tertiary | 91 (16.9%) |
| Monthly income (ZAR^2^) |  |
| None | 143 (26.5%) |
| <R1000 | 61 (11.3%) |
| R1000-R4000 | 154 (28.6%) |
| R4001-R8000 | 124 (23%) |
| >R8000 | 52 (9.6%) |
| Refused | 2 (0.4%) |
| Unknown | 3 (0.6%) |

^1^ SD = standard deviation

^2^ ZAR = South African Rand currency

**Appendix 2.** Gross domestic product (GDP) price deflator methods for updating point-of-care (POC) viral load (VL) testing cost estimates

Per-client costs from a prior POC HIV VL testing pilot study were updated using the GDP price deflator and new prices for test cartridges. Consumer price index (CPI) table values were obtained from the official World Bank data resource for the base year of 2017, when the POC VL tests were first estimated, and inflated to reflect the reported year of 2022. The 2017 POC VL test cost in USD were first converted to South African Rand (ZAR) using the 2017 South Africa to US exchange rate reported by the World Bank (XR: 13.2). We then adjusted for South Africa-specific inflation using CPI values with the following formula: 2022 Cost = 2017 Costs * (2022 CPI) / (2017 CPI). The final value was then converted back to USD using the 2022 South Africa to US exchange rate (XR: 16.36). All POC VL test costs were inflated using the above method except for the Cepheid POC VL test cartridge which had changed in price separate from inflation.^20^ The current individual test cartridge price of USD $14.90 was inputted into the updated estimation of POC VL test costs.^27^ Costs collected in South African Rand were converted to USD using the World Bank 2022 exchange rate, reflecting when data were collected.^48^ POC VL cost estimates in the pilot study accounted for quality control reagents, POC instrument maintenance, and assumed a 5-year instrument life.

**Appendix 3.** Personnel time and costs

Staff salary costs per minute were calculated through monthly salary divided by total minutes worked, assuming an eight hour workday based on staff interviews and job guidelines. During busy mornings with multiple clients, staff members may start intaking a new healthcare client while waiting the five minutes for the point-of-care (POC) tenofovir (TFV) test results to process. However, this was not common and did not appear to impact the clinic visit times, as clients were staggered and previous clients would be addressed while the next client left the room to collect their urine sample. Counselling was always done one client at a time for privacy.

Healthcare personnel time for the POC TFV test was estimated to take a median time of 7:37 (minutes:seconds) (interquartile range [IQR]: 6:07 – 11:41). This time encompassed the active personnel time needed to load the urine sample (n=15), run the tenofovir test (n=32), and counsel the client based on their test result (n=28). A total of six different staff in the Professional Nurse cadre was observed at varying times and geographic locations. We did not analyze peak and off-peak timing differences as the majority of observations were collected in peak morning hours, when there were clients available to observe, thus making comparisons difficult.

**Appendix 4.** Additional sensitivity analyses

Table 1. Per-test cost of point-of-care (POC) tenofovir across variations in personnel time.

| **Cost Per Patient** | **POC Costs by Personnel Testing Time** | | |
| --- | --- | --- | --- |
| Cost Category | Median time | Q1 time^1^ | Q3 time^2^ |
| Capital costs | 3.71 | 3.71 | 3.71 |
| Clinic consumables | 0.29 | 0.29 | 0.29 |
| Test strip | 6.86 | 6.86 | 6.86 |
| Start-up training | 0.54 | 0.54 | 0.54 |
| Personnel costs for testing and counselling | 1.58 | 1.31 | 2.50 |
| **Total cost per test** | **12.97** | **12.70** | **13.90** |

^1^ Q1 (first quartile) refers to the 25^th^ percentile of time

^2^ Q3 (third quartile) refers to the 75^th^ percentile of time

Table 2. Per-test cost of point-of-care (POC) tenofovir at varying clinic testing volumes, assuming median personnel time, 3% discount rate, and a 10-year useful life for start-up and economic costs.

| **Cost Per Patient** | **POC Costs by Clinic Testing Volume** | | | |
| --- | --- | --- | --- | --- |
| Cost Category | 10 | 20 | 50 | 100 |
| Capital costs | 7.28 | 3.64 | 1.46 | 0.73 |
| Clinic consumables | 0.29 | 0.29 | 0.29 | 0.29 |
| Test strip | 6.86 | 6.86 | 6.86 | 6.86 |
| Start-up training | 0.57 | 0.29 | 0.11 | 0.06 |
| Personnel time for testing and counselling | 1.58 | 1.58 | 1.58 | 1.58 |
| **Total cost per test** | **16.58** | **12.65** | **10.30** | **9.51** |
